# Vascular targeted photodynamic therapy: A review of the efforts towards molecular targeting of tumor vasculature

**DOI:** 10.1142/s1088424619300180

**Published:** 2019-12

**Authors:** Vida Mashayekhi, Charlotte Op ‘t Hoog, Sabrina Oliveira

**Affiliations:** a Division of Cell Biology, Department of Biology, Faculty of Science, Utrecht University, Padualaan 8, 3584 CH, Utrecht, The Netherlands; b Pharmaceutics, Department of Pharmaceutical Sciences, Faculty of Science, Utrecht University, Universiteitsweg 99, 3584 CG, Utrecht, The Netherlands

**Keywords:** photodynamic therapy, targeted photosensitizer, vascular targeting, tumor vasculature

## Abstract

The therapeutic value of vascular targeted photodynamic therapy (VTP) for cancer has already been recognized in the clinic: TOOKAD^®^ has been clinically approved in Europe and Israel for treatment of men with low-risk prostate cancer. When light is applied shortly after intravenous administration of the photosensitizer, the damage is primarily done to the vasculature. This results in vessel constriction, blood flow stasis, and thrombus formation. Subsequently, the tumor is killed due to oxygen and nutrient deprivation. To further increase treatment specificity and to reduce undesired side effects such as damaging to the surrounding healthy tissues, efforts have been made to selectively target the PS to the tumor vasculature, an approach named molecular targeted VTP (molVTP). Several receptors have already been explored for this approach, namely CD13, CD276, Extra domains of fibronectin (A, B), Integrin αvβ3, Neuropilin-1, Nucleolin, PDGFRβ, tissue factor, and VEGFR-2, which are overexpressed on tumor vasculature. Preclinical studies have shown promising results, further encouraging the investigation and future application of molVTP, to improve selectivity and efficacy of cancer treatment. This strategy will hopefully lead to even more selective treatments for many cancer patients.

## Introduction

Photodynamic therapy (PDT) involves three components to create local cytotoxicity: a light-activatable drug, named a photosensitizer (PS), light with a specific wavelength that can activate the PS, and oxygen. Separately, the three components are harmless. Together, the light-activated PS will transfer energy to oxygen, forming cytotoxic reactive oxygen species (ROS), which can cause cytotoxicity due to the damage to biomolecules such as lipids, proteins, and nucleic acids. Consequently, the produced ROS can kill cancer cells, damage tumor vasculature, and can also induce inflammatory and immune responses.

The PDT procedure is carried out in two steps: first, the PS is administered, and this is followed by sitespecific light exposure [1]. The degree in which cancer cells or tumor vasculature are preferentially killed can be influenced by varying the time between PS administration and illumination, named the drug-light interval (DLI). When a PS is administered systemically in the body, it is first present in the vasculature. Most commonly in the clinic, a 2 to 4 days DLI is employed to favor the accumulation of the PS in the tumor. This has been described to be driven by the enhanced permeability and retention (EPR) effect. This phenomenon is caused by the unorganized structure of tumor vasculature, enabling macromolecules to extravasate into the extravascular space [2]. In addition, lymphatic drainage is impaired, causing the macromolecules to be retained longer in tumor tissue. Thus, a long DLI provides a strategy to preferentially damage tumor cells using PDT, without any molecular-targeted approach, which we here refer to as conventional PDT. Alternatively, using a short DLI preferentially damages tumor-associated vessels, as the PS is still present in the vasculature, which has been named vascular-targeted PDT (VTP) [3]. VTP mainly damages tumor vasculature, causing vessel constriction, blood flow stasis, and thrombus formation [4]. This vascular shutdown blocks the oxygen and nutrient supply to the tumor and therefore causes necrosis of the tumor.

VTP is a promising therapeutic approach and is expected to have several advantages compared to conventional PDT. First, very hydrophobic PSs are used in conventional PDT, which are retained in the body for longer periods of time in order to accumulate in the tumor. The longer a PS is present in the body, the longer patients can experience photosensitivity after treatment [5, 6]. Second, although light is applied locally in conventional PDT, surrounding healthy tissues are in many cases damaged due to poor tumor selectivity of the PS. Third, lack of oxygen in some areas of the tumors due to the acute hypoxia impairs production of the cytotoxic ROS [7]. Alternatively, in the tumor vasculature, oxygen is more readily available and the vasculature is directly accessible to the PS.

The therapeutic value of VTP for cancer has already been proven in the clinic. In November 2017, the first PS Palladium-metalated bacteriopheophorbide, known as TOOKAD^®^ (Negma Lerads/Steba Biotech), has been approved in Europe and Israel for the treatment of men with low-risk prostate cancer [8]. This treatment can cause fast vascular occlusion and subsequently cancer cell death, within a 5 mm range of the optic fibers. Currently, a phase II clinical trial is ongoing in the US, in which TOOKAD^®^ is applied for the treatment of intermediate risk prostate cancer (NCT03315754). The water-soluble derivative of TOOKAD^®^ (WST11), is now under evaluation in phase I clinical trials for the treatment of esophagogastric cancer (NCT03133650) and upper tract urothelial carcinoma (NCT03617003).

Although VTP is already used in the clinic, efforts have been made beyond this approach to increase selectivity and efficacy of the therapy. For a more general overview of different approaches employed to increase PDT selectivity, the reader is referred to [9]. More selective accumulation of the PS at the vasculature of the tumor would improve the efficacy of the treatment and reduce the risk of side effects. Severe side effects can occur due to PS accumulation in non-targeted tissues such as surrounding nerves and muscles or the bladder and the rectum in particular cases of prostate cancer. In the past two decades, efforts have been made to render the PS specific to the tumor vasculature, namely by targeting particular tumor endothelial markers, a strategy here described as molecular-targeted VTP or molVTP [10]. In this review, we describe research studies that have been performed in the last 15 years, focused on the molecular targeting of PSs to the tumor vasculature for more cancer-specific PDT.

## Molecular Targeting of Tumor Vasculature

**Fig. 1:**
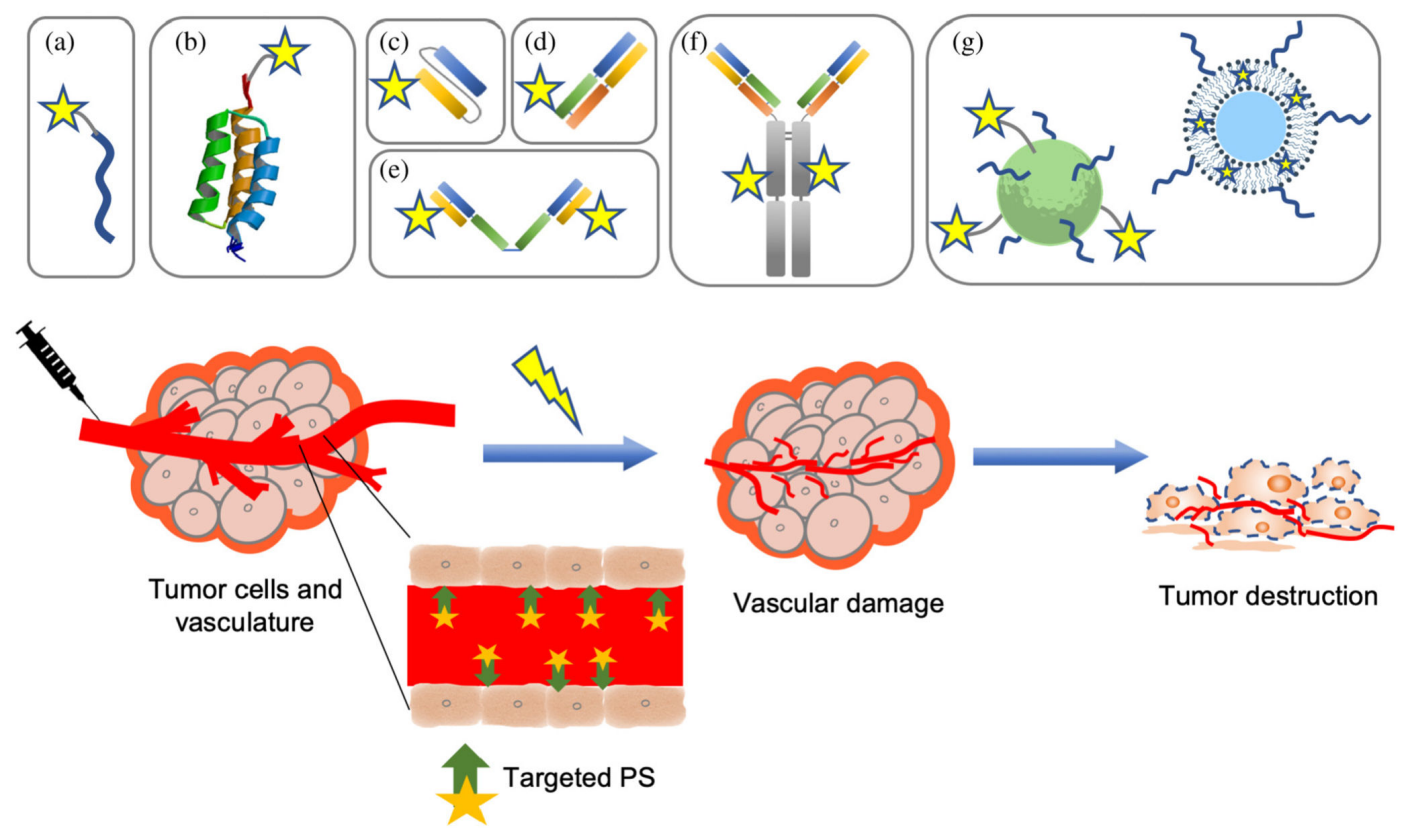
Schematic representation of molVTP and of the different targeting moieties that have been investigated to selectively target PS to the tumor vasculature, for improving selectivity and efficacy of cancer treatment: (a) small peptide, (b) affibody, (c) single chain variable fragment (scFv), (d) antigen-binding fragment (Fab), (e) small immunoprotein (SIP), (f) antibody (IgG), and (g) nanocarrier systems

**Fig. 2:**
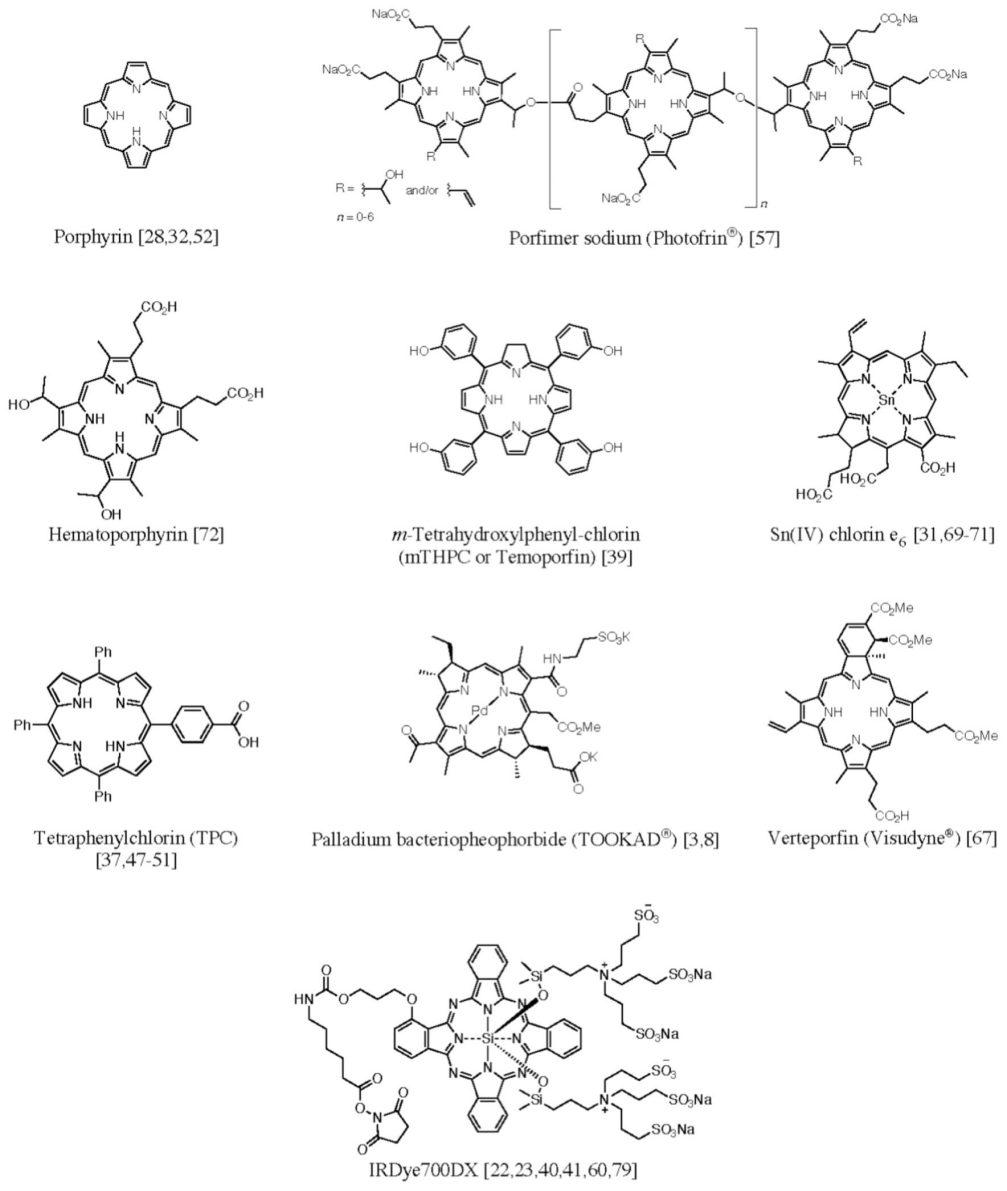
Overview of the chemical structures of the PSs used in the studies discussed in this review

Certain proteins are only present or more abundant on tumor vasculature, which allows for the usage of different types of targeting moieties, such as peptides, antibodies or antibody fragments, and nanocarrier systems, to deliver the PS specifically to the tumor vasculature for molVTP [11] (Fig. 1). Importantly, some of these targets are described to be present not only on tumor-associated vasculature but also on cancer cells, thereby also leading to tumor-cell targeted PDT, or molecular-targeted PDT (molPDT) [12–14]. Even though molecular targeting of tumor cells has also developed considerably over the last decade, such studies are outside the scope of this review. It is nevertheless important to highlight that when both tumor cells and tumor vasculature are targeted, the corresponding effects may be difficult to separate in preclinical studies. In the following sections, the proteins or markers that have been explored for molVTP are described in alphabetical order, and the overview of the chemical structures of the PSs employed is given in Fig. 2.

### CD13/aminopeptidase N

CD13/aminopeptidase is an ubiquitous enzyme which exists in a variety of human cell types (epithelial, endothelial, fibroblast, leukocyte) [15]. This receptor has a role in several biological processes, such as cell proliferation, secretion, invasion and angiogenesis [16]. The overexpression of this receptor in tumor cells and angiogenic vessels, makes it a potential target for molecular targeted therapies.

The peptides containing asparagine-glycine-arginine (NGR) were found to bind selectively to CD13 on tumor vasculature *in vivo* [17]. However, only one study described the application of the NGR peptide as a targeting moiety in the context of PDT [18]. The PS diadduct malonic acid C60 (DMA-C60) was conjugated to NGR peptide and applied *in vitro* in human MCF-7 breast cancer cells. The cells were irradiated for 1 h using a light intensity of 60 mW/cm^2^. Cell viability was significantly decreased and the highest concentration (10 μg/mL) caused a decrease in viability to 20%. Although NGR is potentially a suitable moiety to target tumor vasculature, no studies have been conducted on the effects of DMA-C60-NGR on endothelial cells or *in vivo* models.

### CD276

The transmembrane glycoprotein CD276 is an immuno-regulatory molecule overexpressed on endothelial cells of tumor vasculature and in different types of tumors, while limited expression is seen in normal cells [19, 20]. The physiological functions of CD276 are still unclear [21]. Due to the high level of expression in cancer and endothelial cells, CD276 could be a potential target for combined molVTP and molPDT. In a recent study conducted by Bao *et al*., the Fab fragment of an anti-CD276 antibody (αCD276/Fab) was conjugated to the PS IRDye700-*N*-hydroxysuccinimide (IR700) and the efficacy after PDT was determined in 4T1 tumor-bearing mice [22]. First, it was shown that 4T1 cells and tumor vasculature overexpress CD276, using immunofluorescence staining with an αCD276 antibody, conjugated to Fluorescein (FITC). The FITC-staining colocalized with a marker of 41T cells (integrin β6) and a marker of vasculature (CD31). Thereafter, the tumor uptake of the αCD276/Fab-IR700 was determined *in vivo* in mice subcutaneously xenografted with 4T1 cells. A similar conjugate with irrelevant affinity was used as a control. The uptake was expressed as the percentage of fluorescence intensity, by normalizing uptake values to the total dose. The uptake of the αCD276/Fab-IR700 and the control conjugate was 10% and 4%, respectively. Thus, targeting of CD276 enhanced the uptake of the PS conjugate. Subsequently, the PDT efficacy was determined in 4T1 tumor-bearing mice by measuring tumor growth for 16 days after the treatment. PDT was performed using a light dose of 70 J/cm^2^ and a DLI of 2 h. The targeted conjugate significantly inhibited tumor growth between days 8 to 16, in contrast to the control conjugate. It was already known that 4T1 cells metastasize to lungs when subcutaneously xenografted [23], and in control mice, lung metastases were evident on day 16. However, when the primary xenograft tumor was treated with the αCD276/Fab-IR700, the lungs showed substantially less metastatic foci and weighted significantly less than control lungs. The αCD276/Fab-IR700 conjugate was able to inhibit the formation of lung metastasis by recruiting the tumor infiltration of CD8^+^ T cells in treated mice. The data suggested that PDT targeted to CD276 can potentially be employed to selectively kill tumor vasculature and directly or indirectly tumor cells. The vascular effects of the conjugate were not investigated further, and more studies are needed to determine the utility of αCD276/Fab-IR700 in molVTP.

### Extra domains of fibronectin

Among several splice variants of fibronectin, extra domain A and B (ED-A and ED-B) have been reported to be potential targets for molVTP [10]. Many angiogenic processes, such as invasion, migration, and proliferation of vascular cells are regulated by a number of cell-surface and extracellular adhesion molecules. Fibronectin as an extracellular matrix (ECM) component, expressed in a variety of normal tissues. However, ED-A and ED-B expression is undetectable or negligible in normal adult tissues [24–26].

#### Extra domain A (ED-A)

Using an ED-A specific antibody named F8, Rybak and co-workers found that ED-A is strongly expressed in the neovasculature of human lung and liver metastases, as well as multiple other human tumors, whereas in normal tissues the ED-A expression was negligible [26]. Furthermore, a comprehensive study of human lung tumors showed that ED-A is abundantly expressed in all important subtypes of lung cancer. In order to target this neovascular marker, a small immunoprotein (SIP) of the monoclonal F8 antibody was developed [27]. The SIP(F8) was conjugated to the PS5-[4-(succinimide-*N*-oxycarbonyl)phenyl]-10,15,20-tris-(4-*N*-methylpyridimiumyl)porphyrin trichloride (Tri-PyPhSUCCMeCl^-1^) and microcirculatory effects of the conjugate mediated PDT were assessed by intravital microscopy in mice xenografted subcutaneously with human SF126 glioma cells in a dorsal skinfold chamber [28]. molVTP was performed using a light dose of 150 J/cm^2^ and a DLI of 12 h. The results demonstrated that ED-A-targeted molVTP led to hypoperfused areas in the center of the tumor as a result of stasis and thrombosis. In contrast, the hypoperfused areas were surrounded by hyperperfused areas in the tumor periphery starting 48 h after PDT. This could be the result of molVTP-induced hypoxia, which initiates angiogenesis [29]. When molVTP was applied only once, the hyperperfused areas led to recovery of the tumor vascular circulation, and therefore the anti-tumor effects were only temporary. Importantly, repetitive illumination (12, 24, 36, and 48 h after injection of the conjugate) resulted in sustained reduction of glioma growth after 5 days of observation. The positive effects after repetitive illumination might be attributed to long-lasting vascular dysfunction and lower recovery capacity. Even though the authors suggested Tri-PyPhSUCCMeCl^-1^-SIP(F8) conjugate as a promising candidate in molVTP, in a clinical context, repetitive PDT might be inconvenient for the patients.

#### Extra domain B (ED-B)

Interestingly, the ED-B domain is highly conserved in different species, with 100% homology between human, mouse and rat. Therefore, the same targeting moieties can be used in the clinic and in preclinical studies, which can expedite the translational step to the clinic [30].

A human IgG antibody called L19 has been developed which binds the ED-B domain with high affinity. In a study conducted by Borsi *et al*., biodistribution of the different formats of L19 antibody, *i*.*e*. single-chain variable fragment (scFv) and SIP, was compared to determine which format is the most suitable for targeting applications. The results showed that the SIP might be preferable for a number of tumor targeting applications, compared to scFv and complete antibody (IgG), due to the most suitable clearance rate and *in vivo* stability in tumor-bearing [30].

Afterwards, the same group conjugated scFv(L19) and SIP(L19) to the PS bis(triethanolamine)Sn(IV) chlorin e_6_ (SnChe_6_) [31]. The molVTP efficacy of the conjugates was compared in 3 different subcutaneous tumor models: FE8 sarcoma, F9 teratocarcinoma, and C51 colon adenocarcinoma. PDT was performed using a light dose of 150 J/cm^2^ and a DLI of 5 h for scFv(L19) and 24 h for SIP(L19). Seven days after treatment, the tumors were collected and weighed. In FE8-bearing mice, untreated control tumors weighed on average 1.03 g, while the SnChe_6_-scFv(L19)- and SnChe_6_-SIP(L19)-treated tumors weighed about 0.14 g and 0.04 g, respectively. As SIP(L19)-SnChe6 was suggested to be the most potent, further analysis was continued with this format only. SIP(L19)-SnChe_6_ conjugate caused an arrest in tumor growth, in all three models. In addition, tissue sections of the FE8 tumors were analyzed under the microscope, which showed completely occluded vessels in the SIP(L19)-SnChe_6_-treated tumor. This suggested that the growth arrest happened due to the rapid blood coagulation.

In another study, SIP(L19) was conjugated to a porphyrin-based PS (5-[4-(succinimide-*N*-oxycarbonyl) phenyl]-10,15,20-tris-(4-methylpyridimiumyl)-porphyrin trichlorideand) and anti-cancer effects of molVTP were assessed in 2 models grafted subcutaneously in mice, either F9 murine teratocarcinoma cells or A431 human epidermoid carcinoma cells [32]. molVTP was performed using a light dose of 60 J/cm^2^ after 24 h and 48 h post intravenous injection. Selective accumulation of the conjugate around the tumor vessels caused a selective disruption of the tumor neovasculature after illumination and, subsequently, inhibited long-lasting tumor growth (100 days post treatment). Furthermore, natural killer cells were shown to be essential elements for induction of long-term anti-tumor responses. The findings of this study supported molVTP as a promising strategy to inhibit tumor growth.

### Integrin αvβ3

The effects of the ECM on cell survival, proliferation and differentiation are primarily mediated by integrins, which are heterodimeric transmembrane glycoprotein receptors [33]. Multiple integrins are involved in angiogenesis, but especially αvβ3 integrin has a key role in endothelial survival and migration. Furthermore, αvβ3 integrin is widely expressed on tumor neovasculature, but not on the vasculature of healthy tissues, which makes it a very suitable receptor for molVTP [34].

In 1987, an RGD (arginine, glycine and aspartate) peptide sequence was discovered as the cell attachment site in fibronectin [35]. This sequence was found to be present in many natural ligands of αvβ3 integrin receptors [36]. Unsurprisingly, this RGD peptide was conjugated to different PSs in several studies in order to explore its potential as a tumor-vasculature-targeting moiety for molVTP. Importantly, αvβ3 integrin receptor expression is also found in multiple types of cancer cells. Therefore, the RGD peptide is also widely studied as a potential targeting moiety for tumor-cell-targeted PDT, or molPDT. However, in this review, only the vascular-targeting potential of RGD is discussed.

In the first paper which explored the vascular-targeting potential, linear RGD and cyclic RGDfK peptides were synthesized *via* a solid-phase approach and then conjugated to 5-(4-carboxyphenyl)-10,15,20-triphenylchlorin (TPC) [37]. The targeting potential of the conjugates was tested *in vitro* by comparing the uptake in HUVEC cells overexpressing αvβ3 integrin, with the murine EMT-6 mammary carcinoma cells lacking αvβ3 integrin. Results showed that both conjugates accumulated on average 5 times more in HUVEC cells than in EMT-6 cells. Subsequently, authors showed that the PDT efficacy was higher in HUVEC when using the conjugated PS, compared to the free PS, with comparable results obtained for the two forms of peptide. No phototoxicity was in fact observed in HUVEC nor in EMT-6 cells treated with the free PS.

It has been known that small cyclic peptides are more resistant to proteolysis and have the ability to bind with higher affinity to integrin receptors [38]. Another approach that has been explored to increase the avidity for αvβ3 integrin binding and slow down the rapid washout of RGDfK from tumor vasculature, is by increasing the number of RGDfK peptides per PS/particle. A study by Haedicke and co-workers described the conjugation of the RGDfK peptide to silica-modified calcium phosphate nanoparticles (NPs), decorated with the PS temoporfin (mTHPC) and fluorescent molecule DY682-NHS for near-infrared fluorescence (NIRF) optical imaging [39]. The PDT potency using the NP-DY682-mTHPC was determined in mice subcutaneously xenografted with human CAL-27 tongue-squamous epithelium carcinoma cells. PDT was performed using a light dose of 100 J/cm^2^ and a DLI of 24 h. The reduction in tumor vascularization was assessed (up to 4 weeks after treatment) by measuring a reduction of fluorescence intensity of the contrast agent IRDye^®^ 800CW RGD. Two days after PDT, apoptosis was detected in the tumor and the strongest reduction in tumor vascularization occurred 1 week after the treatment. Tumor volume was significantly reduced in three out of four mice. However, in one mouse, the outer tumor area significantly started to grow 2 weeks after the treatment. According to the authors, a possible explanation for this is the heterogenous distribution of the PS and the incomplete coverage of the tumor during illumination.

This strategy of increasing the number of RGDfK peptides per PS was also studied by Dou *et al*. in the context of PDT [40]. They conjugated the PS IR700 and cyclic RGDfK (cRGD) to a polymer with a polyethylene glycol-poly L-glutamic acid (PEG-PGlu) backbone. The resulting conjugates contained 5 cRGD peptides (IR700-PEG-PGlu-cRGD5) or 15 cRGD peptides (IR700-PEG-PGlu-cRGD15). In addition, a control with 1 cRGD peptide (IR700-PEG-cRGD) and a control lacking affinity for αvβ3 (IR700-PEG-PGlu-RAD15) were used. The distribution of the conjugates was compared *in vivo* in mice subcutaneously xenografted with U87 cells. The IR700-PEG-PGlu-RAD15 conjugate and the free PS did not show tumor-specific accumulation. The monomeric IR700-PEG-cRGD conjugate slightly improved the accumulation. In comparison, the accumulation of IR700-PEG-PGlu-cRGD5 and IR700-PEG-PGlu-cRGD15 were substantially higher, indicating that multiple peptides enhanced the avidity for binding to αvβ3 integrin. Furthermore, the IR700-PEG-PGlu-cRGD5 and IR700-PEG-PGlu-cRGD15 accumulated preferentially within tumor cells and tumor neovasculature, respectively. These results showed that the distribution of the conjugates can be controlled by varying the number of cRGD peptides. Using U87 tumor-bearing mice, the potency of the conjugates was determined *in vivo*. PDT was performed using a light dose of 100 J/cm^2^ and a DLI of 3 h. No statistical difference in tumor growth inhibition was observed between the free PS, IR700-PEG-PGlu-RAD15 and the monomeric 700DX-PEG-cRGD. On the other hand, IR700-PEG-PGlu-cRGD5 and IR700-PEG-PGlu-cRGD15 significantly reduced tumor growth, of which the effect of IR700-PEG-PGlu-cRGD15 was stronger, although the tumor accumulation was similar at the time of the illumination. This suggests that the preferential accumulation in tumor neovasculature improves PDT efficacy. In conclusion, the study showed that by fine-tuning the number of cRGD peptides on this particular system, the avidity for binding αvβ3 integrin can be improved and the PS distribution can be modified.

In another study conducted by Li *et al*., multiple cRGD peptides and PS IR700 were conjugated to human serum albumin (HSA) molecules (cRGD-PEG-HSA-IR700) [41]. Albumin is the most abundant circulating protein in blood and has already been successfully used as a drug carrier, due to its biodegradability and safety profile [42]. Furthermore, albumin has high affinity for secreted protein acidic and rich in cysteine (SPARC), which is often overexpressed in cancer’s extracellular matrix [42–44]. Therefore, this characteristic can potentially improve the affinity of the nanoconjugates. The *in vitro* cellular uptake study showed a 121-fold increase in uptake of the targeted cRGD-PEG-HSA-IR700 nanoconjugates into human TOV21G ovarian cancer cells (αvβ3 overexpressing cells) compared to non-targeted control nanoconjugates. Moreover, cRGD-PEG-HSA-IR700 selectively induced cell death in TOV21G cells with EC_50_ values of ~10 nM, while NIH3T3 cells (no αvβ3 expression) were not affected. Furthermore, the results of live/dead staining confirmed induction of a strong phototoxicity in SKOV-3 spheroids treated with targeted nanoconjugates, indicating penetration of the conjugate into the 3D spheroid model.

### Neuropilin-1

Neuropilin-1 (NRP-1) is known to play a role in neural development, cell survival, migration, angiogenesis, and invasion and it has been implicated in the vascularization and progression of tumors [45].

A comprehensive analysis of NRP-1 expression in human cancer (consisting of 65 primary breast carcinomas, 95 primary colorectal adenocarcinomas, 90 primary lung carcinomas, and 59 metastases) in 98–100% of all the tumor sections, overexpression of NRP-1 was observed in the tumor-associated vessels [46]. NRP-1 overexpression was also detected in some tumor cells such as breast, prostate, and melanoma cells [12], which indicates using a NRP-1-specific moiety could lead to both tumor cell and tumor vasculature targeting. Consequently, several studies have been conducted exploring the potential of NRP-1 for cancer-cell targeted PDT (molPDT) and molVTP.

Tirand *et al*. conjugated a heptapeptide (ATWLPPR) to the PS tetraphenylchlorin (TPC) *via* 6-aminohexanoic acid (Ahx) linker. The PDT efficacy was determined in HUVEC cells 24 h after incubation with free TPC or TPC-Ahx-ATWLPPR. Treatment with free TPC showed little photodynamic activity, whereas HUVECs treated with TPC-ATWLPPR had 10.4-fold lower viability. In nude mice subcutaneously xenografted with human U87 glioma cells, 2.3% of the injected TPC-Ahx-ATWLPPR per gram of tumor tissue accumulated in the tumor at 1 h, and 2.2% at 6 h after intravenous administration. Furthermore, immunohistochemical analysis of the tumor sections revealed that this accumulation at the tumor site is predominantly in the neovasculature. Due to the expression of NRP-1 in U87 tumor cells, this targeting strategy may potentiate tumor cell and molVTP *in vivo* [47]. The *in vivo* molVTP efficacy of TPC-Ahx-ATWLPPR was investigated later by Bechet *et al*., in the same U87 glioblastoma mouse model. PDT was performed using a light dose of 120 J/cm^2^ and a DLI of 4 h [48]. TPC-Ahx-ATWLPPR treatment led to a significant decrease in tumor tissue blood flow compared to treatment with free TPC. Following the expression of tissue factor immediately post treatment, thrombi formation was observed, which resulted in blood vessels’ congestion. Moreover, the induced vascular shutdown caused a significant tumor growth delay compared to the control group. In order to improve the *in vivo* stability of the peptide moiety, Thomas *et al*. created a peptidase-resistant pseudopeptide of ATWLPPR [49]. This was performed by replacing the amide bond between amino acid A and T by –CH_2_NH– bond. The resulting peptide (Aψ[CH_2_NH]TWLPPR) was coupled to the PS TPC *via* Ahx linker. The results of MALDI-TOF mass spectrometry confirmed no degradation of the conjugate, TPC-Ahx-Aψ[CH_2_NH]TWLPPR up to 4 h after intravenous injection in mice.

Benachour *et al*. developed silica-based gadolinium oxide NPs encapsulating the PS TPC and the surface-localized conventional ATWLPPR peptide. The gadolinium oxide core of the NPs was used as a MRI contrast agent. Human MDA-MB-231 breast cancer cells over-expressing the vascular neuropilin-1 (NRP-1) receptor were used to analyze *in vitro* PDT efficacy. The light dose that is able to kill 50% of the cells (LD_50_) for NP-TPC-ATWLPPR-treated cells was determined for two different PS concentrations: 9.16 J/cm^2^ (0.1 μM PS) and 2.80 J/cm^2^ (1.0 μM PS). In contrast, no significant cytotoxicity was observed in the cells treated with non-targeted NPs. Therefore, the results showed that NP-TPC-ATWLPPR is a selective and potent agent for therapeutic and imaging purposes [50]. Importantly, the results of the *in vivo* study in rats bearing an orthotopic U87 glioblastoma demonstrated selective accumulation of targeted NPs in the endothelial cells of tumor vessels after intravenous injection.

Later, the same group developed other peptides targeting NRP-1, of which two were selected for further investigation, named DKPPR and TKPRR [51]. The advantage of these peptides is that they show higher displacement of the ligands of NRP-1, compared to ATWLPPR. The two peptides were conjugated to TPC *via* Ahx linker, PEG9 or PEG18 (the number refers to the number of PEG molecules) as a spacer, to ensure some distance between TPC and the peptide. The conjugation was done by a solid-phase approach to gain specific conjugation of the PS to the amino-terminal of the peptides. The stability and distribution of the conjugates were then investigated in healthy mice [51]. DKPPR conjugates showed better tissue distribution and plasma stability; therefore this peptide was chosen for the production of NRP-1-targeted AGuIX NPs (polysiloxane-based) containing the PS TPP and gadolinium as contrast agent [52]. In order to graft the targeting peptide on the surface of the NPs *via* a solid-phase approach, an additional lysine (K) was added, resulting in KDKPPR as atargeting moiety. To assess NPs distribution in the tumor, U87 cancer cells were implanted in a skinfold chamber in nude mice. The intravital microscopy results showed selective localization of the targeted AGuIX-TPP-KDKPPR in tumor vasculature 1 h post injection and remained visible for 24 h, in contrast to the untargeted AGuIX-TPP which was found free in the blood vessels and removed after 6 h. In order to evaluate PDT *in vitro*, HUVEC cells were treated with AGuIX-TPP-KDKPPR or AGuIX-TPP for 4 h and then cell survival was measured after illumination (5 or 10 J/cm^2^). Cells treated with targeted-NPs and illuminated with light doses of 5 and 10 J/cm^2^ caused 50% and 98% cell death, respectively, while untargeted NPs induced low toxicity.

### Nucleolin/C23

Nucleolin/C23 is mainly located in the nucleus and plays a role in modulation of cellular progression. However, the expression of this protein is significantly increased in many types of cancers and also mainly localized on the cell surface of both tumor and tumor-associated endothelial cells [53]. High expression of cell surface nucleolin participates in cell adhesion, migration, and invasive behavior [54]. A fragment of the human high-mobility group protein 2 (HMGN2), called vascular homing peptide 3 (F3), has been shown to bind to cell surface nucleolin and holds cell-penetrating properties [55, 56].

Reddy *et al*. reported the application of iron oxide NPs with the F3 peptide in rats with 9L rat glioma cells orthotopically implanted in the brain [57]. The NPs contained the PS Photofrin and iron oxide as contrast agent for imaging. PDT was performed using a DLI of 24 h. When compared to the non-targeted NPs or free Photofrin, rats treated with the targeted NPs exhibited a significantly enhanced overall survival. The median survival of untreated mice, mice treated with the non-targeted NPs, or the targeted NPs was 7.0, 13.0 and 33 days, respectively. Out of the 5 mice treated with targeted-NPs, 2 mice were still disease free 6 months after treatment. These survival rates are promising, thus further studies are awaited to explore the efficacy of these NPs in molVTP.

The F3 peptide can potentially be explored for tumor-targeted PDT strategies as well, as NPs coated with F3 peptide have shown to give specific targeting to selected tumor cells *in vitro*, including 9L, MDA-MB-435, and F98 [58].

### Platelet-derived growth factor receptor β

Thus far, only markers on endothelial cells have been discussed as potential targets for molVTP. However, it is known that the walls of tumor vasculature predominantly consist of irregularly lined endothelial cells and pericytes [59]. Consequently, pericyte-targeted PDT has also been explored, namely through targeting of the platelet-derived growth factor receptor β (PDGFRβ) [60]. PDGFRβ is a tyrosine kinase receptor, crucial for the development of kidney, lung and cardiovascular system in the embryo. This receptor is overexpressed on the pericytes of many types of tumors, including lymphomas, colon, ovarian, prostate, lung, and breast cancers. The stimulation of PDGFRβ has been shown to increase the coverage of the tumor vessels and subsequently to improve vessel function. Moreover, in some cases the activation of this receptor increased tumor growth rates [61].

To target PDGFRβ, a dimeric Z_PDGFRβ_ affibody was developed [60]. Affibody molecules are nonimmunoglobulin-derived affinity proteins based on a three-helical bundle protein domain [62]. The dimeric Z_pcGFRβ_ affibody was conjugated to the PS IR700 (Z_IR700_) and tested *in vivo* in mice subcutaneously xenografted with human LS174T colorectal cancer cells. molVTP was performed using a light dose of 120J/cm^2^ and a DLI of 4 h. Tumor grafts were removed and weighed at different time points and HIF1α-expressing cells were visualized using confocal laser scanning microscopy with an anti-HIF1α antibody. The expression of HIF1α was significantly increased in tumor tissue after treatment with Z_IR700_, which reflects tumor hypoxia. In addition, histological staining revealed that blood clots were present in tumor tissue 4 h after treatment with Z_IR700_ and light, in contrast to the control in the absence of light. Thus, the results showed that targeting pericytes can cause hypoxia in the tumor due to thrombosis. The average mass of mice tumor grafts after treatment with Z_IR700_ was approximately 20–30% of that of the mice treated with PBS or Z_IR700_ in the absence of light. This indicates that vascular damage can inhibit tumor growth and pericyte-targeted PDT can be an efficient approach in cancer treatment. Importantly, some cancer cells such as several types of ovarian and breast cancers also express PDGFRβ [63, 64] and therefore using the Z_PDGFRβ_ affibody as targeting moiety can potentially be used in the combination of molPDT and molVTP.

### Tissue factor

Yet another potential target for molVTP is Tissue Factor (TF). TF is a transmembrane glycoprotein, which plays an important role in hemostasis and thrombosis. In normal conditions, active TF is not expressed but when damage of the vascular wall happens, subendothelial TF is expressed/exposed to blood circulation and binds to plasma factor VIIa [65]. In cancer, TF is expressed by active macrophages, stromal cells, and tumor-associated endothelial cells, which have been described to contribute to metastasis, tumor growth, and angiogenesis [66].

Hu *et al*. were the first group to describe targeting of a PS to TF using factor VII (fVII is a natural ligand of TF). In this study, murine fVII (mfVII) was conjugated to the PS verteporfin *via N*’-3-dimethylaminopropyl-*N*-ethylcarbodiimide hydrochloride (EDC) linker and the potency was determined *in vitro* in murine EMT6 and human MDA-MB-31 breast cancer cells. Results of *in vitro* PDT, using a light dose of 60 J/cm^2^ with TF-targeted verteporfin, showed three to four times more potency than the free verteporfin. In addition, the specificity for angiogenic endothelial cells was assessed using *in vitro* PDT, with a light dose of 36 J/cm^2^, in HUVEC cells in the presence and absence of VEGF. No statistical difference was observed in the viability of angiogenic and quiescent HUVEC treated with free verteporfin. In contrast, mfVII-verteporfin caused a significant decrease in viability in angiogenic HUVEC cells, but not in quiescent HUVEC [67]. In addition to angiogenic endothelial cells, many types of tumor cells, such as breast cancer cells, also overexpress TF [68]. Therefore, fVII-targeted PDT could be employed for combined molPDT and molVTP, possibly having a broad therapeutic potential for cancer treatment. Later, the PDT efficacy was determined in mice subcutaneously grafted with murine breast cancer EMT6 cells. The PDT treatment was performed 4–6 times with an interval of 2 or 3 days, using a light dose of 105 J/cm^2^ and a DLI of 90 min. Free verteporfin did not have any effect on tumor growth, while mfVII-targeted PS was effective in inhibiting tumor growth over 18 days of observation.

Due to the high cost of extraction of verteporfin from Visudyne^®^, the same group conjugated mfVII to Sn(IV) chlorin e_6_ (SnChe_6_) [69]. *In vitro* PDT was performed in MDA-MB-231 cells, using a light dose of 36 J/cm^2^. Results showed that TF targeting enhanced the ability of SnChe_6_-mediated PDT to kill MDA-MB-231 cells 12-fold, with LD_50_ values (dose killing 50% of cells) of PS concentration of 0.58 μM and 7.00 μM for targeted and non-targeted SnChe_6_, respectively. In addition, the effect of TF expression level on the efficacy of targeted PDT was determined by treating human MDA-MB-231 (high TF expression), MCF-7 breast cancer cells (low expression of TF) and 293 cells (no TF expression). It was found that the phototoxicity was directly correlated to the expression level, and that 293 cells were not affected. These results suggest that TF-targeted PDT can selectively kill TF-expressing cells without damaging surrounding cells. The mfVII-SnChe6 conjugate was also tested *in vivo* in mice subcutaneously grafted with murine EMT6 cells or human MDA-MB-231 cells. PDT was performed using a light dose of 72 J/cm^2^ and a DLI of 90 min. EMT6 tumors treated with mfVII-SnChe_6_ weighed significantly less than that those from control mice (*p* < 0.05). In the MDA-MB-231 model, even though targeted PDT significantly inhibited tumor growth, the differences in tumor weight were not statistically significant (*p* > 0.05).

The same group investigated the efficacy of SnChe_6_-mfVII-targeted PDT *in vitro* and *in vivo* in lung cancer models [70]. Besides SnChe_6_-mfVII, they also developed a mfVII-SnChe_6_ conjugate containing two repeats of the nuclear localization sequence (NLS), forming mfVII/ NLS-SnChe_6_. The efficacy of targeted PDT using a light dose of 36 J/cm^2^ was determined *in vitro*, which showed that TF-targeting could enhance the killing of A549 and H460 cells up to 25-fold in comparison to the free PS. Furthermore, mfVII/NLS-SnChe_6_ was slightly more effective than mfVII-SnChe_6_ (without NLS). Subsequently, mfVII/NLS-SnChe_6_ conjugate was used for further investigation *in vivo* in mice subcutaneously xenografted with A549 cells. Mice were treated with PDT twice a week for six weeks using a light dose of 120 J/cm^2^ and a DLI of 90 min. Results showed that the TF-targeted SnChe_6_ can significantly inhibit tumors, in contrast to the free PS. Together, these studies suggest that TF-targeted SnChe_6_ could be a suitable treatment modality for targeting tumor neovasculature and cancer cells, namely lung and breast cancer cells.

The specificity of TF for angiogenic vessels has been verified by Hu *et al*. [71]. Using ELISA, they tested the expression of TF on primary human vascular endothelial cells derived from three major types of human vessels: microvascular, umbilical venous, and aortic endothelial cells. TF expression was determined in the presence of VEGF as an *in vitro* model for angiogenic vessels and in the absence of VEGF as an *in vitro* model for quiescent vessels. Results showed that TF is specifically expressed on all three types of angiogenic vessels and not on quiescent vessels. Furthermore, fVII selectively bound angiogenic endothelial cells but not quiescent endothelial cells, using a cell ELISA experiment. In addition, it was shown that TF-targeted PDT, using fVII-SnChe_6_ with a light dose of 36 J/cm^2^ only induced apoptosis and necrosis in angiogenic endothelial cells. The non-targeted PDT using free SnChe_6_ had no detectable effect on either angiogenic or quiescent endothelial cells. Thus, results suggested that fVII could be used to selectively target a PS to angiogenic vessels such as tumor neovasculature.

In a different study, PEG-PLGA NPs were loaded with the PS Hemoporfin (hematoporphyrin monomethyl ether) and coupled to EGFP-EGF1 [72]. EGFP-EGF1 is a fusion protein derived from fVII which contains the specific TF binding capacity [73]. First, the NPs uptake was determined in rat brain capillary endothelial cells (BCEC), which were stimulated by tumor necrosis factor-a (TNF-a) to induce TF expression. The targeted NPs accumulated significantly more in the BCECs than non-targeted NPs. Moreover, the TF expression after PDT was determined with western blotting and real-time PCR. Results showed that the targeted NPs led to increased expression of TF in BCECs after PDT. In addition, the cellular ROS level was determined using fluorescence microscopy and showed higher levels of intracellular ROS in BCECs treated with targeted-PDT, suggesting induction of TF expression by ROS. The EGFP-EGF1-targeted NPs were further studied in a mouse model xenografted subcutaneously with human CA46 Burkitt lymphoma cells. The tumors were harvested 24 h post PDT and stained for TF expression and NPs distribution. Observation of tumor sections using confocal laser scanning microscopy showed that the targeted NPs accumulated more in the tumor vasculature than in other parts of the tumor tissue and that PDT increased TF expression. Altogether, the results suggested that PDT using EGFP-EGF1-NPs containing Hemoporfin is a suitable strategy for molVTP. Importantly, the strategy employed a positive feedback loop, which enhanced the targeting of the NPs: the existing TF on neovasculature was used to target the NPs to the tumor, and as a result of the ROS production after PDT, it is suggested that more TF expression is induced, enabling more accumulation of targeted NPs in the tumor vessels.

### Vascular endothelial growth factor receptor 2

The vascular endothelial growth factor receptor 2 (VEGFR-2) is a 200 kDa glycoprotein which belongs to the tyrosine kinase family. This receptor plays an essential role not only in physiological angiogenesis from early embryonic to adult stages, but also in pathological angiogenesis such as cancer [74]. This receptor is activated by its ligand VEGF, which is a mitogen for endothelial cells [75]. VEGF is upregulated in response to hypoxia and many oncogenes, which in turn upregulates the expression of VEGFR-2 in vascular endothelial cells [76, 77]. This receptor is less abundant in normal blood vessels and highly expresses in tumor vasculature. The significant difference in expression level makes this receptor an ideal target for selective delivery of therapeutics to tumor neovasculature [78]. However, few research groups have explored its application in molVTP in the last 15 years.

Recently, Nishimura *et al*. compared vascular and tumor-targeted photoimmunotherapy (PIT) in a mouse model. In this study, the PS IR700 was conjugated to DC101, a monoclonal antibody targeting murine VEGFR-2, and to trastuzumab, a monoclonal antibody against human HER2. Mice xenografted subcutaneously with HER2 overexpressing human NCI-N87 gastric cancer cells and treated with either conjugates showed anti-tumor effects, although the therapeutic effect of DC101-IR700 was suggested to be the strongest and to be mediated by a decrease in tumor microvessel density [79]. Due to the upregulation of VEGFR-2 in different cancers, the authors suggested the application of this strategy for treatment of various types of cancer. Further studies are awaited exploring VEGFR-2 targeting for molVTP.

## Conclusion

This review provides an overview of the targeting moieties which have been explored to provide PS selectively to tumor vasculature in order to reduce side effects and increase treatment efficacy. As described, different conjugates have been developed for molVTP, ranging from small peptides, affibodies (~7 kDa), to intermediate molecular size, as scFvs (~30 kDa), Fabs (~50 kDa), SIPs (~80 kDa), to larger IgGs (~150 kDa), and nanocarrier systems (Fig. 1). In general, a larger size of the moiety causes a decrease in tumor penetration and in clearance rate, while it can increase immunogenicity. The right balance is of importance to achieve optimal PDT efficacy. However, the properties of the PS affect this balance substantially, and differences in distribution and accumulation can therefore not be solely attributed to the type of targeting moiety that is used.

Different strategies have been described to increase the size or delay the clearance from the tumor vasculature. For instance, the size can be increased by adding polymers such as polyethylene glycol. Furthermore, targeting moieties can be conjugated or grafted on nanocarrier systems, *e.g.* liposomes, or on endogenous drug carriers such as albumin. Another example is the study in which 1, 5 or 15 RGDfK peptides were conjugated to IR700. The conjugate containing 5 peptides accumulated preferentially in the tumor, while the conjugate containing 15 peptides accumulated preferentially in the vasculature. Thus, by modifying the conjugates, the properties can potentially be optimized.

Among the described conjugates, the vascular effects after molVTP have explicitly been shown *in vivo* for NRP-1-targeting verteporfin-ATWLPPR and TPC-ATWLPPR conjugates, ED-B-targeting SnChe_6_-SIP(L19) and por-phyrin-SIP(L19), ED-A-targeting TriPyPhSUCCMeCl^-1^-SIP(F8), αvβ3 integrin-targeting calcium phosphate NPs with mTHPC and RGDfK peptides, and pericyte-targeting PDGFRβ-IR700. Therefore, these conjugates can be considered in a more developed stage of preclinical molVTP research.

Concerning clinical translation, the DLI is of particular relevance. The conjugates described in this review required DLIs from 90 min to 24 h. When a long DLI is required for optimal molVTP efficacy, and when this is due to a very hydrophobic PS, the risk of side effects and phototoxicity could be higher. Only two conjugates caused regression of the tumor, in contrast to tumor inhibition observed in the rest of the *in vivo* studies. This concerns the calcium phosphate NPs with mTHPC and RGDfK peptides and the iron oxide NPs with Photofrin and the F3 peptide. Despite the positive results, both formulations required a DLI of 24 h, which could discourage its clinical application. On the other hand, the tumor growth inhibition and vascular effects were explicitly shown with TPC-ATWLPPR and the PDGFRβ-IR700 conjugates, which required a relatively short DLI of 4 h.

In addition to the parameters described above, the expression level of the target certainly contributes to PDT efficacy. The more it is overexpressed at the tumor vasculature, and the less the target expresses elsewhere in the body, the higher the molVTP efficacy will be and the fewer the chances of damaging surrounding tissues. Furthermore, some targets (αvβ3 integrin, nucleolin, NRP-1, ED-A and TF) also overexpress on certain types of tumor cells. Therefore, depending on the tumor type, one conjugate can be preferred over another due to the expression on both tumor neovasculature and tumor cells. This would enable a dual-targeting PDT strategy, which overall is expected to enhance treatment efficacy.

Even though some targets have been more elaborately investigated compared to others, it is still too early to predict which one will first be evaluated in the clinical setting. Due to the variable experimental parameters (type of PS and targeting moiety or formulation, light dose, size of tumor, DLI, *etc*.) in the different studies, it is hard to draw a solid conclusion regarding the most promising target and targeting moiety for molVTP. Likely, this will vary per tumor type or even perhaps per patient.

Further preclinical studies are needed to investigate the vascular effects of the conjugates and the feasibility for clinical application. Although molVTP conjugates have not been used in any clinical trials yet, the results of preclinical studies are promising, indicating potential application of molVTP to improve selectivity and efficacy of cancer treatment.
